# Opportunities and challenges in the treatment of IgA nephropathy

**DOI:** 10.3389/fphar.2025.1559593

**Published:** 2025-07-30

**Authors:** Yang Yang, Gaosi Xu

**Affiliations:** ^1^ Department of Nephrology, The Second Affiliated Hospital, Jiangxi Medical College, Nanchang University, Nanchang, China; ^2^ Jiangxi Key Laboratory of Molecular Medicine, The Second Affiliated Hospital of Nanchang University, Nanchang, China

**Keywords:** immunoglobulin A nephropathy, galactose-deficient IgA1, treatment, nefecon, sparsentan

## Abstract

The treatment paradigm of immunoglobulin A nephropathy (IgAN) is shifting, and traditional therapeutic strategies are insufficient to meet clinical needs. Based on the increasing understanding of the pathogenesis of IgAN, current treatment goals concentrate on anti-inflammatory and targeted therapy, as well as optimizing therapy. New therapeutic approaches are being developed, including complement inhibitors, B-cell activating factor and a proliferation-inducing ligand inhibitor, and endothelin receptor antagonists. Further supportive care showed promising prospects and combination therapy such as sodium-glucose cotransporter 2 inhibitor with endothelin receptor antagonists are also being investigated, which may provide greater benefit. IgAN is a disease that requires lifelong management, the treatment choices faced may be inconsistent at different ages and periods. With the emerging opportunities in IgAN treatment, achieving individualized precision therapy is a key challenge currently facing research institues. This review summarizes recent advances in the treatment of IgAN and discusses possible therapeutic strategies for IgAN patients.

## 1 Introduction

Immunoglobulin A nephropathy (IgAN), the most common primary glomerulonephritis in the world, has an overall incidence of at least 2.5 per 100,000 people annually, with a higher prevalence in Asians than in non-Asians (Kiryluk et al., 2023; Lee et al., 2023). Recently study revealed that 27% IgAN patients experienced renal failure during a median follow-up of 3.3 years (Sim et al., 2025). Combined with high prevalence and poor prognosis of IgAN, the 2024 KDIGO draft revised the therapeutic goal to proteinuria <0.5 g/d, ideally <0.3 g/d, and estimated glomerular filtration rate (eGFR) decreasing <1 mL/min per year.

Previously, the main treatments of IgAN is focus on optimized supportive care, including blood pressure management, maximally tolerated dose of renin-angiotensin system inhibitors (RAASi), lifestyle modification and address cardiovascular risk (Kidney Disease: Improving Global Outcomes Glomerular Diseases Work, 2021). Many patients with residual proteinuria after treatment with maximal doses of RAASi are considered to be at high risk for progressive chronic kidney disease (CKD), and these patients require immunosuppressive therapy (Kidney Disease: Improving Global Outcomes Glomerular Diseases Work, 2021). Systemic corticosteroids (CS) are a treatment method that limit glomerular inflammation, but associated with severe infection and impaired glucose tolerance (Rauen et al., 2015; Lv et al., 2022), its clinical benefit is remain controversial. At present, the first widely approved specific treatment for IgAN, nefecon, through targeting the release of budesonide to clear galactose-deficient IgA1 (Gd-IgA1) (Barratt et al., 2024b). Based on the understanding of the upstream mechanism of Gd-IgA1 production, additional therapeutic strategies are being explored, such as targeted B-cell activators, plasma cell depleting agents, and proteasome inhibitors. Complement activation has been reported in IgAN (Duval et al., 2023; Medjeral-Thomas et al., 2021), and targeting different pathways of complement activation similarly adds new therapeutic strategies.

The challenge of IgAN lies in its typically asymptomatic nature, often diagnosed when concerns arise due to macroscopic hematuria or proteinuria, at which time some patients may already have evidence of CKD and face the loss of their functioning nephrons (Rodrigues et al., 2017; Caster et al., 2023). Furthermore, IgAN exhibits a highly variable clinical course, with significant heterogeneity observed between children and adults. The inflammatory responses is more pronounced in the former, and there are also certain differences in treatment (Antonucci et al., 2024). Infections is one of the factors that may trigger disease progression, thus necessitating individualized precision therapy. In the face of the current emergence of new therapeutic drugs, it is necessary to continuously optimize anti-inflammatory, targeted therapies and supportive care in order to reduce proteinuria and prevent renal function decline. The focus of this article is on the paradigm shift in the treatment of IgAN to enhance understanding of the different therapy options.

## 2 Treatment of IgAN

**FIGURE 1:**
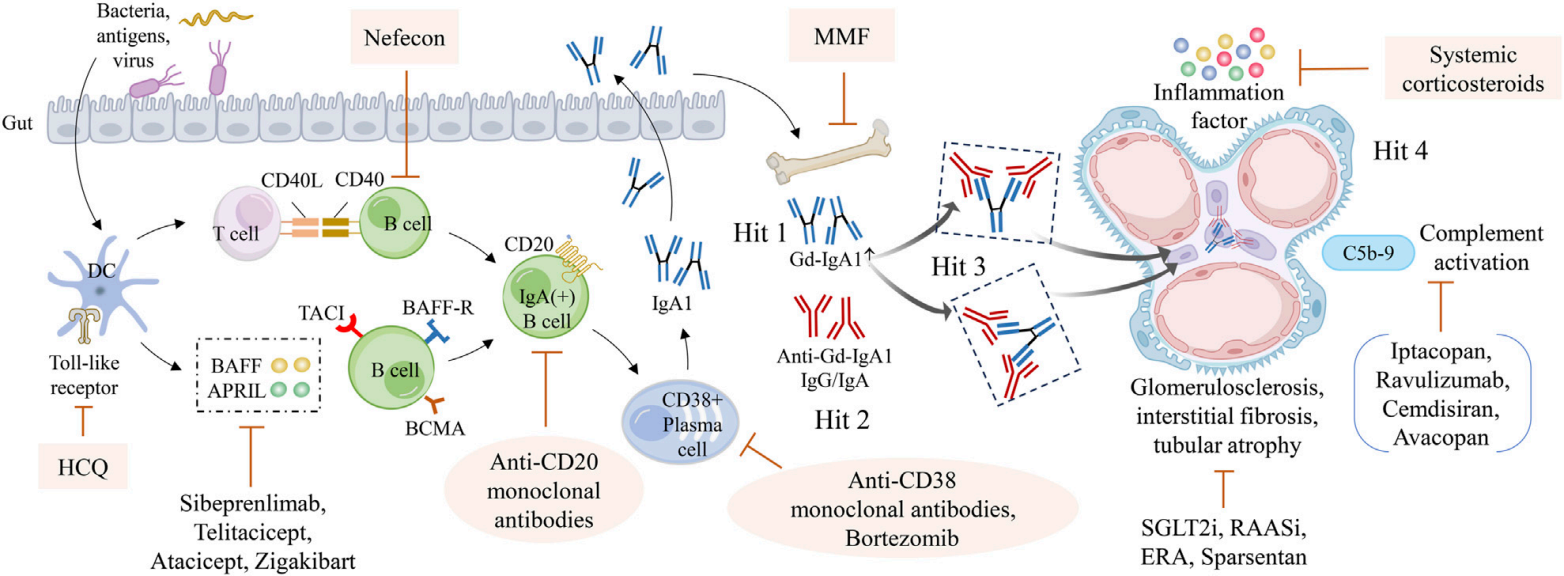
The pathogenesis of IgAN and each drugs therapeutic targets. Hit 1, the excessive generation of Gd-IgA1; Hit2, the formation of anti-Gd-IgA1 antibodies; Hit 3, the formation of circulating immune complexes; Hit 4, the circulating immune complexes deposit in the glomerular mesangium. Different drugs act on different targets to delay disease progression. ERA: endothelin receptor antagonists; HCQ: hydroxychloroquine; Gd-IgA1: galactose-deficient IgA1; MMF: Mycophenolate Mofetil; RAASi: renin-angiotensin-aldosterone system inhibitors; SGLT2i: sodium–glucose cotransporter-2 inhibitor.

The current understanding of the pathogenesis of IgAN focus on the “multi-hit” hypothesis. Combination of genetic and environment factors (infections, food antigens, microbiological disorders) lead to the activation of mucosal immune cells via Toll-like receptors, where they secrete large amounts of BAFF and APRIL cytokines. These cytokines stimulate B-cell activation, proliferation and IgA class switching in a T-cell-independent manner. Activated B-cells traffic to central lymph nodes and then traffic back to mucosal sites or mis-trafficking to the bone marrow (Selvaskandan et al., 2024a). Mucosally-derived plasma cells produce Gd-IgA1. In circulation, Gd-IgA1 was recognized by autoantibodies and formation the immune complexes deposited in the glomerular mesangial areas, causing glomerular inflammation and ultimately progressive kidney dysfunction (Cheung et al., 2024a). Various therapies for different targets have emerged (Figure 1).

### 2.1 Supportive pharmacologic therapy

Unlike other glomerular diseases, maintaining hemodynamic homeostasis remains the cornerstone of IgAN. Over the past several decades, the optimized supportive treatments, such as RAASi, which effectively lower intraglomerular pressure, inhibit inflammatory, and reduce proteinuria, have been verified to slow the progression of IgAN (Figure 1) (Praga et al., 2003; Li et al., 2006). In the DAPA-CKD and EMPA-KIDNEY trials, IgAN patients treated with novel antidiabetic drug sodium-glucose cotransport 2 inhibitors (SGLT2i) experienced a reduction in cardiovascular and renal failure events (Table 1) (Wheeler et al., 2021; Herrington et al., 2023), but it remains unclear whether SGLT2i is equally effective in patients with preserved renal function.

**TABLE 1:** Recently therapeutic agents published for IgAN.

Treatments (NCT number)	Target	Study design (intervention)	Follow-up	Main outcomes			
				Proteinuria	eGFR	Safety	Other
Anti-inflammatory therapies							
Immunosuppression (NCT00554502)	Systemic	Multicenter, open-label, RCT T: supportive care plus immunosuppression C: supportive care	36 months	4 patients (6%) achieved UP < 0.2 g/g in the control group while 14 (20%) in treatment group	No difference in mean absolute change	No difference in total AEs, but higher severe infections, glucose intolerance, weight gain in treatment group	36 (50%) patients in treatment and 35 (45.5%) in control group had all-cause death, ESRD, or eGFR loss >40% after 10 years
Corticosteroids (NCT01560052)	Systemic	Multicenter, RCT T: methylprednisolone 0.6–0.8 mg/kg/d for 2 months, tapering by 8 mg/day each month C: placebo	12 months	24-h UP was lower in the treatment group, between-group difference of 0.69 g/d, not apparent by 3 years	Annual rate of eGFR loss was 2.50 (treatment) and 4.97 mL/min/1.73 m^2^/year (control)	Serious AEs were more frequent (10.9% vs 2.8%) and dose-related in the treatment group	No difference in efficacy between the full-dose and reduced-dose of methylprednisolone
Corticosteroids	Systemic	Retrospective cohort study Full-dose group: corticosteroids 0.8–1.0 mg/kg/d for 2 months, tapered by 20% each month for the next 4 months; half-dose group: corticosteroids 0.4–0.6 mg/kg/d for 2 months, tapered by 20% each month for the next 4 months plus RAASi	18 months	UP reduction in the half-dose and the full-dose group at 6 months were 1.39 g/d and 1.44 g/d, respectively	One patient in half-dose group and three patients in full-dose group had eGFR decrease >30%	AEs was lower in the half-dose group	No difference in complete remission and overall remission rate at 6 and 18 months
Iptacopan (NCT04578834)	CF B	RCT, phase 2 Part 1: iptacopan 10, 50, and 200 mg twice daily or matching placebo for 180 days Part 2: iptacopan 10, 50, 100, and 200 mg twice daily or matching placebo for 270 days	6 months	23% reduction in UPCR from baseline in the iptacopan 200 mg group compared with placebo at 3 months	eGFR decreased by 3.34 mL/min/1.73 m^2^ in the placebo group and remained stable in all iptacopan groups at 3 months	Well tolerated, no deaths, serious AEs or bacterial infections	Plasma Bb and urinary sC5b-9 levels were reduced in the iptacopan group
Iptacopan (NCT04578834)	CF B	Multicenter, RCT, phase 3 T: oral iptacopan 200 mg twice daily C: placebo	9 months	24-h UPCR was 38.3% lower in the iptacopan group than in the placebo group	No data	No difference in total AEs	Urine C5b-9 restored to within healthy levels in the iptacopan group
Ravulizumab (NCT04564339)	C5	Multicenter, RCT, phase 2 T: ravulizumab intravenous every 8 weeks plus supportive care for 26 weeks C: placebo; all participants received open-label ravulizumab for 25 weeks	50 weeks	24-h UP was lower in the treatment group, between-group difference of −30.1% at week 26	Annualized eGFR slope was −2.34 (treatment) and −4.09 mL/min/1.73 m^2^/year (placebo)	No difference in total AEs	Percentage of patients with absence of hematuria increased over time with ravulizumab treatment
Cemdisiran (NCT03841448)	Small interfering RNA-targeting C5	Multicenter, RCT, phase 2 T: cemdisiran 600 mg subcutaneous every 4; weeks C: placebo	32 weeks	24-h UPCR was lower in the treatment group, between-group difference of −37.4%	Annualized eGFR slope was lower in the treatment group, between-group difference of 5.14 mL/min/1.73 m^2^/year	No difference in total AEs	Hematuria and serum C5 levels were reduced more in the treatment group
Avacopan (NCT02384317)	C5aR1	Open-label pilot trial, phase 2 Avacopan 30 mg twice daily for 12 weeks, then received RAASi only for 12 weeks	24 weeks	Reduction in the UPCR in 6/7 patients (∼50% in 3/7) at week 12	Remained stable	Well tolerated	Both urine red blood cells and urinary monocyte chemoattractant protein-1 were reduced
Narsoplimab (NCT02682407)	MASP-2	Single-arm open-label study Substudy 1: narsoplimab 4 mg/kg infusion once-weekly for 12 weeks plus steroid Substudy 2: T: narsoplimab 370 mg C: placebo	18 weeks/91 weeks	Substudy 1: 4 patients with an overall median reduction of 72% to baseline Substudy 2: UP reductions in 18.0% (control) and 18.4% (treatment)	eGFR levels remained stable in both substudys	Well tolerated	-
Targeting pathogenic IgA1 synthesis							
Nefecon (NCT03643965)	Targeted-release	Multicenter, RCT, phase 3 T: nefecon 16 mg/d C: placebo	24 months	Time-averaged UPCR decreased by 40.9% relative to baseline in the nefecon group and increased by 1.0% in the placebo group	Time-weighted average change of eGFR was lower in the nefecon group, between-group difference of 5.05 mL/min/1.73 m^2^	Well tolerated, no difference in total and severe AEs	Occurrence of a 30% reduction in eGFR or renal failure at a later time in nefecon group
Sibeprenlimab (NCT04287985)	APRIL	Multicenter, RCT, phase 2 T: sibeprenlimab 2, 4 or 8 mg/kg intravenous C: placebo	12 months	24-h UPCR was reduced by 47.2%, 58.8%, 62.0%, and 20.0% in sibeprenlimab 2, 4, 8 mg, and placebo groups	Annualized eGFR slope estimate was attenuated in sibeprenlimab groups as compared with placebo	No difference in total AEs	Gd-IgA1 and APRIL levels were reduced in the sibeprenlimab 4-mg and 8-mg groups
Zigakibart (NCT03945318)	APRIL	Open-label, two cohort study BION-1301 450 mg once intravenous every 2 weeks	12 weeks	UP reduced	No data	Well tolerated	Gd-IgA1 reduced
Zigakibart (NCT03945318)	APRIL	Cohort 1:450 mg Q2W, 24 weeks, then 600 mg Q2W Cohort 2: 600 mg Q2W	100 weeks	24-h UPCR was reduced by 60% in both cohorts combined	Sustained eGFR stabilization, with an annual eGFR slope of +0.48 mL/min/1.73 m^2^	Well tolerated	74% in Gd-IgA at week 76, 74.3% reductions in IgA and 80% in IgM at week 100
Atacicept (NCT04716231)	BAFF + APRIL	Multicenter, RCT, phase 2b T: atacicept 25, 75, or 150 mg C: placebo	36 weeks	Combined 150 mg and 75 mg atacicept reduced average UPCR by 24% at week 24, 35% at week 36 vs. placebo	Mean eGFR between-group difference of 11%	Well tolerated, no difference in total AEs	Combined 150 mg and 75 mg atacicept reduced Gd-IgA1 by 60% from baseline vs placebo
Atacicept (NCT04716231)	BAFF + APRIL	Multicenter, RCT, phase 3, open-label Atacicept 150 mg weekly	96 weeks	UPCR reduced by −52%–65% from baseline	Mean eGFR annualized slope of −0.6 ± 0.5 mL/min/1.73 m^2^/year	Well tolerated, no difference in total AEs	Reduction in Gd-IgA1 from baseline of −66% ± 2%; hematuria patients decreased by −75%
Telitaticept (NCT04291781)	BAFF + APRIL	RCT, phase 2 T: telitaticept 160, 240 mg C: placebo	24 weeks	Telitacicept reduced UP by 0.889 g/d (240 mg group) and 0.316 g/d (160 mg group)	Telitacicept increased mean eGFR by 2.34 (240 mg) and 4.32 mL/min/1.73 m^2^ (160 mg), vs a decrease of 5.70 mL/min/1.73 m^2^ with placebo	No difference in total AEs	Serum levels of IgA, IgG, and IgM decreased in the treatment groups
Bortezomib (NCT01103778)	Proteasome	RCT Bortezomib 1.3 mg/m^2^ per dose for 4 doses	12 months	3/8 patients achieved UP < 0.3 g/d	No data	Well tolerated	3 patients with minimal proteinuria and stable renal function for 2–5 years
MMF + corticosteroids (NCT01269021)	Systemic	Multicenter, open-label, RCT T: MMF 1.5 g/d for 6 months plus prednisone 0.4–0.6 mg/kg/d for 2 months, tapered by 20% each moth for the next 4 months C: prednisone 0.8–1.0 mg/kg/d for 2 months, tapered by 20% each month for the next 4 months	12 months	No difference in complete remission rate (UP < 0.3 g/d) and overall remission rate (UP 0.4–1.0 g/d)	Both groups were higher than baseline values after 2 months of treatment	No difference in AEs, but decreased Cushing syndrome and diabetes in MMF group	Improved in endocapillary hypercellularity, crescents, and necrosis lesions in glomeruli
MMF (NCT01854814)	T cell, B cell	RCT, open-label, blinded end-point T: MMF 1.5 g/d for 12 months, tapered to 0.75 g/d for 6 months plus supportive care C: supportive care	3 years	MMF group had higher UP reduction rate than SC group (57.1% vs. 28.2%)	11 (12.9%) in MMF group, 30 (35.3%) in control group had eGFR reduction >30%	No difference in total AEs and severe AEs	6 (7.1%) in MMF and 18 (21.2%) in control had creatinine doubling, ESRD, death
HCQ (NCT02942381)	Toll-like receptors	RCT, phase 2 T: HCQ C: placode	6 months	UP was lower in the treatment group (−48.4% vs. −10.0%)	No difference in percentage change in eGFR	No difference in total AEs	No difference in percentage change in frequency of hematuria
HCQ (NCT02942381)	Toll-like receptors	Retrospective cohort study HCQ 0.2 g bid (eGFR >60 mL/min/1.73 m^2^) or HCQ 0.1 g tid (eGFR 45–60 mL/min/1.73 m^2^) or HCQ 0.1 g/d (eGFR <30 mL/min/1.73 m^2^)	24 months	UP decreased from 1.69 to 1.00 g/d	No difference in change in eGFR	10 (5.6%) occurred AEs and no severe AEs were recorded	No difference in the blood pressure levels
Therapies addressing the chronic kidney disease							
Dapagliflozin (NCT03036150)	SGLT2	Multicenter, RCT T: dapagliflozin 10 mg C: placode	32 months	Urinary albumin-to-creatinine ratio was lower in the treatment group, between-group difference of −26.0%	Annualized mean squares eGFR slope was lower in the treatment group, between-group difference of 1.2 mL/min/1.73 m^2^/year	No difference in total AEs	Blood pressures were lower in dapagliflozin group
SC0062 (NCT05687890)	ERA	RCT, phase 2 T: SC0062 5, 10, and 20 mg once daily C: placebo	24 weeks	12 weeks: UPCR was reduced by −27.6%, −20.5%, and −38.1% in the SC0062 5, 10, 20 mg dose groups 24 weeks: reduced by −22.4%, −30.9%, and −51.6%	Mean eGFR changes from baseline were 1.1, 2.2, 0.5 mL/min/1.73 m^2^ at week 12	No difference in total AEs	UPCR was consistent in participants using and not using SGLT2 inhibitors
Atrasentan (NCT04573478)	ERA	Multicenter, RCT, phase 3 T: atrasentan 0.75 mg/d C: placebo	36 weeks	UPCR was lower in the treatment group, between-group difference of −36.1%	No data	No difference in total and severe AEs	Mean weight change was −0.2 ± 2.8 kg for atrasentan, −0.1 ± 3.0 kg for placebo
Sparsentan (NCT03762850)	ERA + ARB	Multicenter, RCT, phase 3 T: sparsentan 400 mg/d C: irbesartan 300 mg/d	110 weeks	UPCR was lower in the treatment group (−42.8% vs. −4.4%)	eGFR 2-year chronic slope was lower in the treatment group, between-group difference of 1.1 mL/min/1.73 m^2^/year	No difference in total AEs, but more dizziness and hypotension occurred in treatment group	18 (9%) in treatment and 26 (13%) in control group had all-cause death, ESRD, or eGFR loss >40%

AEs, adverse events; ARB, angiotensin receptor blockers; eGFR, estimated glomerular filtration rate; ESRD, end-stage renal disease; ERA, endothelin receptor type A receptors; HCQ, hydroxychloroquine; IgAN, immunoglobulin A nephropathy; MASP-2, mannan-binding lectin-associated serine proteinase 2; MMF, mycophenolate mofetil; RCT, randomized controlled trial; SGLT2i, sodium-glucose cotransport 2 inhibitor; UP, urine protein; UPCR, urine protein-creatinine ratio.

#### 2.1.1 Endothelin (ET) receptor inhibitors

ET has long been recognized as the most potent and long-lasting vasoconstrictor, components of the ET system are expressed throughout the kidney (Barton and Yanagisawa, 2008), and pathological factors such as proteinuria are capable of increasing the production of renal ET-1 (Kohan and Barton, 2014). By binding to endothelin receptor type A (ETA) receptors, ET-1 causes vasoconstriction, podocyte nephrin shedding, mesangial hyperplasia, tubulointerstitial fibrosis, and glomerulosclerosis (Kohan and Barton, 2014). Clinical trials showed that targeting ETA have a significant antiproteinuric effect in patients with CKD, even under the use of the maximum RAASi (Wenzel et al., 2009; Dhaun et al., 2009).

SC0062 is a novel and selective ETA antagonist with a favorable safety, tolerability and pharmacokinetic profile in healthy adult volunteers, laying the foundation for the study of SC0062 in renal disease (Liu et al., 2024). One study observed that compared to placebo, 24 weeks of treatment with SC0062 at dose of 5, 10, and 20 mg resulted in changes in urine protein-creatinine ratio (UPCR) of −22.4% (−42.2 to 4.3), −30.9% (−48.6 to −7.0), and −51.6% (−64.2 to −34.6), respectively, showing a linear dose response relationship (Heerspink, et al., 2024b). Atrasentan is a potent and highly selective ETA antagonist with an approximate 1800:1 selectivity for ETA to ETB (Opgenorth et al., 1996). It reduces glomerular intracapsular pressure by reducing vasoconstriction in the efferent arteries, and it also reduces proteinuria by inhibiting macrophage infiltration in renal tissues (Sasser et al., 2007) and restoring endothelial glycocalyx (Boels et al., 2016). Phase 3 trial evaluating in patients with high-risk progressive IgAN showed that atrasentan significantly reduced UPCR (by on geometric mean 36.1%) over 36 weeks of the study compared with placebo (Heerspink, et al., 2024c). Although neither study showed an increased risk of peripheral oedema, the risk of sodium retention is not negligible, and interestingly, the combination of SGLT2i with an ETA inhibitor may alleviate this problem (Heerspink et al., 2021).

It is worthing that ET-1 and angiotensin II (Ang II) have similar pathophysiological effects, and there are complex links between the two systems. ET-1 stimulates the formation of Ang II(Barton et al., 1997), in turn, Ang II also activates increase production of renal ET-1 (Kawaguchi et al., 1990), thereby creating a positive feedback loop. Simultaneously blocking Ang II and ET-1 activity not only corrected proteinuria, but also restored the number of podocytes (Gagliardini et al., 2009). PROTECT is a phase 3 study of sparsentan, dual ETA/Ang II receptor type 1 antagonist, enrolling 404 patients with IgAN (Rovin et al., 2023). The results showed that the sparsentan group demonstrated a greater reduction in proteinuria compared to the irbesartan group, achieving a relative reduction of 40% at 110 weeks and the 2-year chronic slope of eGFR was also lower in the sparsentan group (−2.7 vs. −3.8 mL/min/1.73 m^2^/year, *P* = 0.037) (Rovin et al., 2023), indicating a good long-term effect. Numerous studies are under evaluating the efficacy of RAASi, SGLT2i, and endothelin receptor antagonist combination therapy (NCT03762850, NCT05856760, and NCT05834738).

#### 2.1.2 Mineralocorticoid receptor antagonists

Finerenone, a non-steroidal mineralocorticoid receptor antagonist, has clearly improved renal and cardiovascular prognosis in CKD patients and type 2 diabetes mellitus (Bakris et al., 2020; Pitt et al., 2021). Phase 3 trial in patients with non-diabetic CKD are ongoing, in which 417 (26.3%) patients with IgAN were recruited, and if a beneficial effect on slowing the decline in eGFR is established, finerenone may become another option for renal protection in patients with IgAN (Heerspink et al., 2024a).

### 2.2 Anti-inflammatory therapies

#### 2.2.1 Systemic CS

Systemic CS modulate a plethora of target genes and transcription factors, and attenuating the inflammatory response caused by immune complexes deposition in the glomerulus (Cain and Cidlowski, 2017). In the STOP-IgAN trial, 76 and 78 patients in the supportive care and immunosuppression groups, respectively, completed the 3-year trial phase. The latter group had a higher risk of adverse events (AEs) (Rauen et al., 2015). The primary composite endpoints in all-cause death, end-stage renal disease, and eGFR loss exceeding 40% remained indistinguishable between the two groups after an extended follow-up of up to 10 years (Rauen et al., 2020).

In TESTING trial, the proteinuria reduction in the CS group was higher than the placebo group during follow-up, but the difference was no longer apparent by 3 years, and the study had to be stopped in view of several cases of fatal AEs (Lv et al., 2022). Then, this trial reduced CS dosage. Both full-dose and reduced-dose groups achieved consistent results in reducing the risk of renal composite outcomes, while the reduced-dose group remarkably decreased the incidence of AEs (Lv et al., 2022). Difference in the results of the two studies may be attributed to racial difference in the subjects.

A similar comparison was made in a retrospective cohort study of IgAN patients who were treated with half-dose CS plus RAASi or full-dose CS for 6 months (Wang et al., 2019). There was no significant difference between the two groups at 6 months and 18 months in terms of proteinuria reduction and eGFR decrease >30%, but the half-dose group showed a high advantage in AEs (Wang et al., 2019). This CS reduction regimen may be a good option for IgAN patients at high risk of progression.

#### 2.2.2 Targeting the complement system

Complement activation is thought to play an important role in driving IgAN glomerular inflammation and injury. Renal biopsies from up to 90% of IgAN patients show glomerular deposition of C3, which reflect the activation of alternative approaches (Poppelaars et al., 2021). Spontaneous hydrolysis of C3 generates C3a and C3b, C3b cleavage the crucial cofactor factor B in the presence of factor D, leading to the formation of C3 invertase, C3bBb, which drives the activation of the complement cascade reaction (Barratt and Weitz, 2021). Iptacopan, an oral factor B inhibitor, has been shown to reduce proteinuria in a dose-dependent manner in IgAN patients and it was well-tolerated (Zhang et al., 2024). Recent phase 3 results showed that iptacopan resulted in a significant reduction in proteinuria of 38.3% (95% CI, 26.0–48.6) relative to placebo, and urinary C5b-9 in the iptacopan group was also restored to within the range of healthy levels, providing evidence that changes in biomarkers of the complement pathway are consistent with inhibition of the selective alternative pathway (Perkovic et al., 2024). Another clinical trial on RO7434656, an antisense inhibitor of complement factor B is recruiting (NCT05797610). Moreover, factor D, which is responsible for activating of factor B, has also been found to be elevated in IgAN (Poppelaars et al., 2021). Vemircopan is able to inhibit the activity of complement factor D, thereby blocking the initiation phase of the alternative complement pathway and reducing inflammatory damage and disease progression. Vemircopan is currently being evaluated in phase 2 trial in IgAN and proliferative lupus nephritis (NCT05097989).

Complement lectin pathway activation is supported by the deposition of C4, C4d, mannan-binding lectin (MBL), and MBL-associated serine protease 2 and rare C1q depositon (Roos et al., 2006; Nam et al., 2020). Narsoplimab is a fully human monoclonal antibody that binds with high affinity to proenzyme and enzymatically active forms of human MBL-associated serine proteinase 2, preventing the formation of the C3 convertase, C4bC2b (Dudler et al., 2023). As the key effector enzyme of the lectin pathway, MBL-associated serine protease 2 is activated after carbohydrate expressed on the surface of injured cells or pathogens bind to pattern-recognition molecule (Dudler et al., 2023; Barratt et al., 2023). A small study supports the clinically meaningful of narsoplimab in reducing proteinuria and eGFR stability in patients at high risk of IgAN (Lafayette et al., 2020). However, phase 3 study IgAN patients with high baseline proteinuria is terminated due to failure to show positive results (NCT03608033).

The blockade of the terminal common complement pathway, namely, C3, C5 and C5a, was also evaluated in patients with IgAN. Pecetacoplan is a selective inhibition of C3, which is being evaluated in a phase 2 trial (NCT03453619). Eculizumab, a humanized monoclonal antibody that selectively inhibits C5, its use in IgAN is limited to case reports and varies widely (Rosenblad et al., 2014; Ring et al., 2015). Ravulizumab is a second-generation C5 inhibitor designed by eculizumab. Phase 2 study showed statistically significant proteinuria reduction with ravulizumab compared to placebo and lower annualized eGFR slope (Lafayette et al., 2024b). The phase 3 study had a follow-up period of up to 106 weeks to observe its long-term efficacy and safety (NCT06291376). Moreover, cemdisiran is a small interfering RNA conjugated to N-acetylgalactosamine that inhibits C5 synthesis in hepatocytes (Barratt et al., 2024a). Cemdisiran administration was associated with a significant reduction in 24-h UPCR with concomitant reduction in serum C5 levels (Barratt et al., 2024a). Another small study of the selective C5a receptor inhibitor avacopan similarly showed improvement in UPCR (Bruchfeld et al., 2022).

### 2.3 Targeting therapies

#### 2.3.1 Targeting gut mucosa B cell priming

IgA not only the key point in the pathogenic of IgAN, but also the primary immunoglobulin at mucosal surfaces. Most IgA is produced in mucosa-associated lymphoid tissue, with the highest production in gut-associated lymphoid tissue (Cheung et al., 2024a). Growing evidence supports pathogenic microbial infections or environmental factors are the mainly triggers for IgA production in cells within mucosa-associated lymphoid tissue (Cheung et al., 2024a; Selvaskandan et al., 2024b). Signaling molecules released by intestinal microbes activate intestinal epithelial cells and the mucosal immune system, inducing the activation of mucosal B lymphocytes, which complete the IgA class switch and eventually differentiate into IgA-producing cells (Dang et al., 2025). IgAN patients have been found to have disturbed intestinal flora (Gleeson et al., 2024), which may lead to an imbalance in the above processes, such as mucosal infections, microbiota disorder or abnormal immune activation, resulting in the production of large amounts of IgA, including Gd-IgA1 (Liao et al., 2023).

A novel oral, Nefecon, glucocorticoid-targeted release agent, has been developed for delivery in the terminal ileum, which a major site of mucosal IgA production. The budesonide is directed to the mucosa associated lymphoid tissue, where it reduced Gd-IgA1 production by inhibiting mucosal B-lymphocyte activation and Peyer plaque proliferation (Liao et al., 2023). A total of 364 IgAN patients were recruited in the phase 3 study, the results showed that at 24 months, the change from baseline in eGFR was significantly lower in the Nefecon group (−6.11 mL/min/1.73 m^2^) than in the placebo group (−12.00 mL/min/1.73 m^2^) (Lafayette et al., 2023). Although patients in the Nefcon group had a remarkably lower UPCR than those in the placebo group at 24 months, but both groups had an elevated UPCR at 12 months (Lafayette et al., 2023), implying that sustained inhibition of gut-associated lymphoid tissue is required to maintain suppression of pathogenic IgA production and provide renal protect.

#### 2.3.2 Targeting B-cell activators

The formation of circulating immune complexes in IgAN is inseparable on the survival of B-cell and plasma cells that produce Gd-IgA. B-cell activating factor (BAFF) and a proliferation-inducing ligand (APRIL) are key cytokines that stimulate B-cell activation, proliferation and IgA class switching through BAFF-receptor, B-cell maturation antigen or transmembrane activator and calcium modulator ligand interactor (TACI) in a T-cell-independent manner (Vincent et al., 2013; Cheung, et al., 2024b). These cytokines bind to B-cell and further enhance the proliferation of mature plasma cells and promote the secretion of immunoglobins, including Gd-IgA. Accumulate evidence showed that BAFF and APRIL levels are elevated in IgAN and correlate with disease severity, such as mesangial cell proliferation and segmental glomerulosclerosis (Sallustio et al., 2021; Zhai et al., 2016; Xin et al., 2013). Dysregulation of these cytokines decreased serum Gd-IgA1 levels (Mathur et al., 2024; Barratt et al., 2022; Barratt et al., 2024c), thus inhibit the progression of disease.

Sibeprenlimab is a humanized IgG2 monoclonal antibody that binds to and neutralizes APRIL activity. Clinical trial demonstrated that sibeprenlimab suppressed the UPCR and serum level of APRIL in a dose-dependent manner. More stable renal function in the high dose group, but after drug discontinuation, Gd-IgA1 and APRIL gradually returned to baseline levels (Mathur et al., 2024), implying that long-term suppression of APRIL may be required to maintain clinical efficacy. Zigakibart (BION-1301) binds to specific epitopes on April that blocks APRIL. Reductions of proteinuria were observed as early as 12 weeks and was paralleled by a reduction in Gd-IgA1 levels (Barratt et al., 2022). The results of zigakibart in patients with IgAN treated for 100 weeks were recently reported. UPCR decreased by an average of 60.4% at week 100, Gd-IgA1 levels continued to decrease by an average of 74% up to week 76, and importantly, renal function remained stable during the follow-up period (Kooienga et al., 2025).

Dual BAFF and APRIL blockers, atacicept, telitacicept and povetacicept, are in advanced stage of clinical development. They are fusion proteins containing extracellular portions of TACI and crystallizable fragment domain of IgG (Selvaskandan et al., 2024a). By inhibiting the stimulation of BAFF and APRIL on B cells, they disrupted B cell maturation, differentiation and effector functions, thereby blocking the upstream immune pathogenesis of IgAN. Previous double-blind period results showed atacicept significantly reduced UPCR at week 24 and week 36 (Lafayette et al., 2024a). Barratt er al. reported long-term results in these IgAN patients entering the open-label extension of atacicept. Over 2-year treatment period, atacicept 150 mg per week reduced Gd-IgA1 by 66%, hematuria by 75%, and UPCR by 52%, accompanied by stable renal function and a favorable safety profile (Barratt et al., 2024a), suggesting that atacicept offers a potentially safe, long-term treatment option for IgAN. A study involving 44 Chinese patients with IgAN showed that treatment with telitacicept at doses of 240 mg/week and 160 mg/week results in reductions in 24-h proteinuria by 0.889 g/d and 0.316 g/d, respectively, while maintaining stable eGFR and exhibiting good tolerability (Lv et al., 2023). Phase 3 studies on B-cell activator drugs all underway (NCT05799287, NCT05248646, NCT05248659, NCT05852938, and NCT04716231).

#### 2.3.3 Targeting plasma cell

Plasma cells differentiated from IgA1+ B cells can maintain Gd-IgA and anti-Gd-IgA antibodies production. It has been reported that the percentage of plasma cells in Gd-IgA positive B cells is higher in IgAN patients (Zachova et al., 2022), further suggesting the participate of plasma cells.

Felzartamab and mezagitamab are both fully human IgG1 monoclonal antibody, with a high affinity for cluster of differentiation 38 (CD38) (Endell et al., 2012; Smithson et al., 2017). Felzartamab depletes plasmablasts and plasma cells through antibody-dependent cellular cytotoxicity and antibody-dependent cellular phagocytosis (Endell et al., 2012; van de Donk et al., 2018). Specifically, recruitment and activation of natural killer cells by binding to CD38^+^ plasma cells, followed by release of granzyme and perforin leading to lysis and death of CD38^+^ plasma cells (van de Donk et al., 2018). Moreover, felzartamab also promotes phagocytosis by macrophages (van de Donk et al., 2018). Two studies are ongoing examining CD38 depletion with them (NCT05065970 and NCT05174221).

Proteasome inhibitors, bortezomib, a plasma cell depleting agent, it inhibits the ubiquitin-proteasome pathway responsible for the degradation of most intracellular proteins by blocking the activity of the 26S proteasome thereby inducing apoptosis (Tan et al., 2018). The study by Hartono et al. recruited eight IgAN patients, three of who achieved complete remission after 1 year and maintained that efficacy at long-term follow-up (Hartono et al., 2018). However, these three patients had Oxford classification T scores of 0 and low baseline serum creatinine.

#### 2.3.4 Inhibition lymphocyte proliferation

In IgAN, mycophenolate mofetil (MMF) reduces IgA production by B cells via inhibiting T cell activation and lymphokine production. MMF plus half-dose CS was not significantly different in reducing proteinuria compared with full-dose CS, but decreased the incidence of Cushing syndrome and newly diagnosed diabetes mellitus (Hou et al., 2017). This provides clinical evidence for immunosuppressive effects of MMF supplemented with CS. However, no significant difference in adverse effects was also found between MMF plus CS and CS treatment alone in a retrospective study that included Caucasian IgAN patients (Fontana et al., 2023). Recently, Hou et al. observed that MMF monotherapy significantly reduced the renal composite outcomes compared to the supportive therapy alone (Hou et al., 2023). But termination of MMF accelerated the annual rate of decline in eGFR in IgAN patients, suggesting that the beneficial effects of MMF may not last long after discontinuation (Hou et al., 2023). The efficacy of MMF is currently limited to Chinese patients, and differences in efficacy between races and regions remain to be demonstrated.

### 2.4 Other immunosuppressive agents

Activation of the intrinsic immune response is critical to the pathogenesis of IgAN, and toll-like receptors link intrinsic and adaptive immunity. Hydroxychloroquine (HCQ), a potent inhibitor of toll-like receptors-9, reduces B-cell activation in IgAN. A study enrolling 60 IgAN patients described the benefit of HCQ in reducing proteinuria at 6 months follow-up (Liu et al., 2019). Long-term data showed that HCQ combined with RAASi reduced proteinuria by approximately 32.3% within 24 months with no significant decrease in eGFR and no serious AEs (Tang et al., 2021).

*Tripterygium Wilfordii Hook F* is a traditional herb in China, it reduced proteinuria in IgAN patients via anti-inflammatory, balance of Th17 and Tregs, and downregulate IgA class switching (Li et al., 2017; Chen et al., 2015). A small-scale study conducted in China reported that 40 mg of *Tripterygium Wilfordii Hook F* daily significantly increased the response rate in IgAN patients with heavy proteinuria, and without increase AEs (Wang et al., 2017). Our previous studies showed that Kunxian capsule, a novel tripterygium preparation, also significantly reduced proteinuria in IgAN patients (Yang et al., 2025).

In addition, cyclophosphamide, azathioprine, and leflunomide were also reported to treatment of IgAN. Natale et al. concluded that the benefits of these three regimens for IgAN patients remain uncertain (Natale et al., 2020). But, a recent retrospective study observed that cyclophosphamide significantly reduced proteinuria and decreased the incidence of renal endpoint events (Luo et al., 2023). Leflunomide produces anti-inflammatory effects by blocking tyrosine kinase activation, and a single-center study found a significant advantage of leflunomide in reducing hematuria (He et al., 2024). Pervious evidence support combined with azathioprine may be slightly more effective than CS alone in IgAN patients with chronic renal insufficiency (Pozzi et al., 2013). Since these treatments have not demonstrated substantial benefits for the vast majority of IgAN patients, the 2021 KDIGO guidelines do not recommend them substances routinely.

## 3 Personalize IgAN treatment

Unlike adult IgAN, pediatric IgAN present with more hematuria and proteinuria and show more active lesions in their kidney biopsies such as mesangial proliferation and endocapillary hypercellularity (Su et al., 2024). In younger IgAN children, the high incidence of macroscopic hematuria may reflect more inflammatory lesions that may respond better to treatment (Antonucci et al., 2024), and children with IgAN exhibit a stronger repair capacity (Coppo and Thomas, 2020). Retrospective study showed that immunosuppression, especially CS, appears to be beneficial in children with glomerular inflammation and proliferation (Cambier et al., 2018). Compared with adult, children with IgAN are more likely than adults to be treated with CS and to achieve a higher proteinuria remission rate (Su et al., 2024).

**FIGURE 2:**
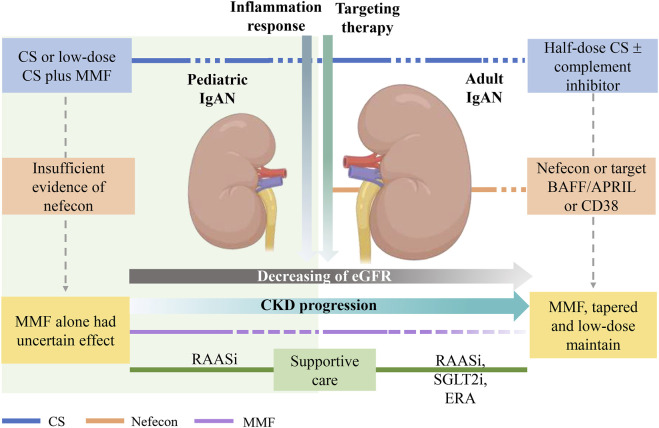
Treatments for adult and pediatric IgAN. In IgAN patients, eGFR decreased progressively with age, and the progression of chronic kidney disease worsen progressively. During the course of IgAN, inflammation response gradually diminishes. Therapeutic goals include anti-inflammatory, targeted therapies, and maintaining hemodynamics stable. For adult patients, high inflammation response requires half-dose CS or complement inhibitor, followed by consideration of the addition of nefecon, BAFF/APRIL, or CD38 inhibitor to reduce pathogenic IgA1. Maintenance low-dose of MMF after gradual tapering may be an adjunctive therapy for Chinese IgAN patients. For pediatric IgAN, CS is beneficial in children with active lesions, and MMF combined with low-dose CS is an alternative option. In addition, CS may be used in low-dose multiple times depending on the condition. There is insufficient evidence on the use of nefecon or MMF alone in children IgAN patients. Targeted therapies may not need to accompany the entire IgAN treatment, whereas supportive therapy need to be received from the onset of the disease, SGLT2i and endothelin receptor antagonists also available for adult patients, their combined efficacy is being evaluated. APRIL, a proliferation-inducing ligand; BAFF, B-cell activating factor; CD38, cluster of differentiation 38, CS, corticosteroids; eGFR, estimated glomerular filtration rate; MMF, mycophenolate mofetil; IgAN, IgA nephropathy; SGLT2i, sodium–glucose cotransporter-2 inhibitors.

Despite the cost in terms of increased risk of infection, CS remain an important weapon for IgAN patients at high kidney risk (Zhang et al., 2023), and the recommended duration of treatment is 6 months. Mucosal immunity is activated in IgAN patients at any age (Ma et al., 2023; Leow et al., 2023). Chronic and acute mucosal infections, as well as other environmental factors, can exacerbate the production of Gd-IgA1 and pathogenic immune complexes (Duval et al., 2023). In these high-risk and relapsing patients, CS may be necessary to reduce glomerular inflammation, and patients may subsequently receive nefecon or BAFF/APRIL inhibitor maintenance therapy to control the initial disease pathway. Keskinis et al. advised CS combined with nefecon therapy, while reducing the dose of both to avoid AEs (Keskinis et al., 2024) (Figure 2). In reducing proteinuria in children with refractory IgAN, telitacicept has demonstrated efficacy similar to that observed in adult studies. The research, which included 11 pediatric IgAN patients, showed an approximately 79% reduction in 24-h proteinuria (Liu et al., 2025). Although Nefecon and BAFF/APRIL inhibitors have not been adequately studied in children, they have the potential to become a second-line therapy for pediatric use.

Of note, the latest research evaluated the therapeutic response of IgAN patients to CS by measuring urine soluble CD163 levels in the urine of TESTING patients before and after CS administration (Li et al., 2024). Compared with the placebo group, both the full-dose and reduced-dose groups exhibited significantly lower levels of soluble CD163 in the urine after 6 and 12 months of treatment. Patients with decreased urine soluble CD163 levels had a lower risk of renal progression events (Li et al., 2024). This result indicates that soluble CD163 can serve as a reliable biomarker for CS treatment and that reduced-dose CS, a regimen that was included in the 2024 KDIGO draft, can be effective. The benefits of CS typically manifest over several months, and we speculate that the duration of half-dose CS use in relapsed patients will be shorter in the future. Complement inhibitors may soon become an alternative to systemic CS for limiting intrarenal inflammation and glomerular injury in IgAN patients (Selvaskandan et al., 2024a).

A single-agent approach is unlikely to be sufficient to prevent disease progression (Barratt et al., 2024c), clinical evidence suggests that although single-agent may reduce proteinuria and slow eGFR loss, symptoms may return after discontinuation (Lafayette et al., 2023; Hou et al., 2023), suggesting that continued production and deposition of immune complexes in the glomerulus may require long-term maintenance therapy, thus multi-targeted therapies are needed to achieve this. Low-dose MMF may be a long-term maintenance therapy for Chinese IgAN patients, and MMF may also be used as an adjuvant therapy in combination with low-dose CS in pediatric IgAN (Kang et al., 2015). However, the efficacy of treatment with MMF alone in terms of reducing proteinuria in children IgAN is uncertain (Alladin et al., 2024). Hemodynamic stability needs to be maintained while administering immunotherapy. SGLT2i and endothelin receptor antagonists are gradually being appreciated. Sparsentan can be used as a basic monotherapy alternative to RAASi or in combination with drugs that modulate immune responses (Trachtman et al., 2024), but it may lead to a higher incidence of hypotension (Rovin et al., 2023), which may limit its use in younger patients.

## 4 Conclusion

IgAN is an autoimmune disease. We are entering a whole new era of IgAN. The development of complement inhibitors, nefecon, BAFF/APRIL inhibitors, SGLT2i, ET inhibitors and aldosterone receptor antagonists has opened up opportunities for the treatment of patients with IgAN. Research on combination treatment options is being intensified and promising preliminary results have been achieved. Personalized treatment is a key challenge in current research.

## Abbreviations

AEs, adverse events; Ang II, angiotensin II; APRIL, a proliferation-inducing ligand; BAFF, B-cell activating factor; CD38, cluster of differentiation 38; CKD, chronic kidney disease; CS, corticosteroids; eGFR, estimated glomerular filtration rate; ET, endothelin; ETA, endothelin receptor type A; Gd-IgA1, galactose-deficient IgA1; HCQ, hydroxychloroquine; IgAN; Immunoglobulin A nephropathy; MBL, mannan-binding lectin; MMF, mycophenolate mofetil; RAASi; renin-angiotensin system inhibitors; SGLT2i, sodium-glucose cotransport 2 inhibitors; TACI, transmembrane activator and calcium modulator ligand interactor; UPCR, urine protein-creatinine ratio.
